# Hyperspectral Imaging in Major Hepatectomies: Preliminary Results from the Ex-Machyna Trial

**DOI:** 10.3390/cancers14225591

**Published:** 2022-11-14

**Authors:** Emanuele Felli, Lorenzo Cinelli, Elisa Bannone, Fabio Giannone, Edoardo Maria Muttillo, Manuel Barberio, Deborah Susan Keller, María Rita Rodríguez-Luna, Nariaki Okamoto, Toby Collins, Alexandre Hostettler, Catherine Schuster, Didier Mutter, Patrick Pessaux, Jacques Marescaux, Sylvain Gioux, Eric Felli, Michele Diana

**Affiliations:** 1 Digestive and Endocrine Surgery, Nouvel Hopital Civil, University of Strasbourg, 67000 Strasbourg, France; 2 University Hospital Institute (IHU), Institut de Chirurgie Guidée par l’image, University of Strasbourg, 67000 Strasbourg, France; 3 Research Institute against Digestive Cancer (IRCAD), 67000 Strasbourg, France; 4 Institut of Viral and Liver Disease, Inserm U1110, University of Strasbourg, 67000 Strasbourg, France; 5 Department of Gastrointestinal Surgery, San Raffaele Hospital IRCCS, 20132 Milan, Italy; 6 Department of Surgery, Istituto Fondazione Poliambulanza, 25124 Brescia, Italy; 7 Department of Pancreatic Surgery, Verona University, 37134 Verona, Italy; 8 Dipartimento di Scienze Medico Chirurgiche e Medicina Traslazionale, Sapienza Università di Roma, 00189 Roma, Italy; 9 Ospedale Cardinale G. Panico, General Surgery Department, 73039 Tricase, Italy; 10 Lankenau Medical Center, Wynnewood, PA 19096, USA; 11 ICube Laboratory, Photonics Instrumentation for Health, 67400 Strasbourg, France; 12 Department of Visceral Surgery and Medicine, Inselspital, Bern University Hospital, University of Bern, 3012 Bern, Switzerland; 13 Department for BioMedical Research, Hepatology, University of Bern, 3012 Bern, Switzerland

**Keywords:** hyperspectral imaging, image-guided surgery, hepatectomy, major hepatectomy, post-hepatectomy liver failure (PHLF)

## Abstract

**Simple Summary:**

Major hepatic resections are associated with higher risk of post-operative complications and post-hepatectomy liver failure. After hepatic pedicle clamping, ischemia-reperfusion injury may sometimes lead to post-hepatectomy liver failure. Early detection or, ideally, intra-operative prediction of liver dysfunction or failure would be essential for timely treatment. Hyperspectral imaging (HSI) is a non-invasive technology that detects the relative reflectance of light at a wavelength between 500 and 1000 nm, allowing the quantification of relevant organic compounds. This is the first clinical application of HSI in a monocentric major hepatectomy series, and the reported results suggest that HSI values could be associated with short-term post-operative outcomes.

**Abstract:**

Ischemia-reperfusion injury during major hepatic resections is associated with high rates of post-operative complications and liver failure. Real-time intra-operative detection of liver dysfunction could provide great insight into clinical outcomes. In the present study, we demonstrate the intra-operative application of a novel optical technology, hyperspectral imaging (HSI), to predict short-term post-operative outcomes after major hepatectomy. We considered fifteen consecutive patients undergoing major hepatic resection for malignant liver lesions from January 2020 to June 2021. HSI measures included tissue water index (TWI), organ hemoglobin index (OHI), tissue oxygenation (StO_2_%), and near infrared (NIR). Pre-operative, intra-operative, and post-operative serum and clinical outcomes were collected. NIR values were higher in unhealthy liver tissue (*p* = 0.003). StO_2_% negatively correlated with post-operative serum ALT values (*r* = −0.602), while ΔStO_2_% positively correlated with ALP (*r* = 0.594). TWI significantly correlated with post-operative reintervention and OHI with post-operative sepsis and liver failure. In conclusion, the HSI imaging system is accurate and precise in translating from pre-clinical to human studies in this first clinical trial. HSI indices are related to serum and outcome metrics. Further experimental and clinical studies are necessary to determine clinical value of this technology.

## 1. Introduction

Liver cancer and metastatic liver disease are a leading cause of cancer mortality worldwide, accounting for more than 700,000 deaths annually [1]. Indications for liver resections have expanded with advances in surgical techniques and chemotherapy, allowing surgeons to approach lesions previously deemed unresectable [2,3]. However, major hepatic resections are technically challenging and associated with the highest risk of adverse post-operative outcomes [4,5]. Almost all major hepatectomies require pedicle clamping to reduce bleeding during liver transection, which results in oxygen deprivation, the catalyst for parenchymal damage. This is further compounded when blood flow is reestablished from ischemia-reperfusion injury (IRI). IRI commonly results in post-hepatectomy liver failure [6,7]; thus, predicting IRI to avoid post-operative liver disfunction is important. Before surgery, evaluations with magnetic resonance imaging (MRI), computed tomography (CT) scan, or sequential hepato-biliary scintigraphy can estimate the volume of the future liver remnant (FLR); adding an indocyanine green (ICG) clearance test can provide a functional assessment [8]. Even combined, these modalities provide a poor snapshot of post-operative conditions [9]. In the operating room, ICG fluorescence imaging, microdialysis, carbon dioxide sensors, and near-infrared spectroscopy (NIRS) can assess real-time perfusion [10,11,12,13,14]. However, they are limited by the need for exogenous dye, expensive commercial equipment, and lack of standardization in interpretating results. On-table ultrasonography (US) plays a critical role in detecting inadequate blood supply or outflow obstruction but is operator-dependent and cannot provide a precise real-time map of liver oxygenation [15]. As a result, current technologies inaccurately estimate perfusion or the complication risk following major hepatectomies. Thus, novel optical imaging technologies that could provide real-time intra-operative feedback on oxygenation and localization of ischemic damage are needed.

Hyperspectral imaging (HSI) is a non-invasive technology that detects the relative reflectance of light at a wavelength between 500 and 1000 nm. The science was originally developed for remote sensing, then successfully applied to military, environmental, geology, agriculture, and global change research, and lately used for quantification of relevant organic compounds [16,17]. Our group recently showed the potential benefits of HSI as an intra-operative tool during image-guided surgery in bowel and liver resections; HSI was able to improve the surgical resection lines via real-time overlay of the hyperspectral image and routine red-green-blue (RGB) captures using augmented reality (HYPER; hyperspectral enhanced reality) [18,19]. Furthermore, the quantification and discrimination of different types of liver ischemia, including arterial or total vascular inflow occlusion, were demonstrated for providing a liver viability score in a pre-clinical model of IRI [20,21]. Moreover, higher values of water within hepatic tissue could be related to IRI and inflammation [22,23]. Given these promising preliminary pre-clinical results, the next step for validation was to translate the optical imaging system into the clinical setting.

The goal of this work was to validate a relationship between HSI parameters and post-operative outcomes after major hepatectomy in human subjects. The hypothesis was that the imaging system would be safe, accurate, and precise in translating from pre-clinical to clinical liver resections.

## 2. Materials and Methods

### 2.1. Study Design

The present study was part of the EXMachyna3 project (Intraoperative EXamination Using MAChine-learning-based HYperspectral for diagNosis & Autonomous Anatomy Assessment, Strasbourg, France), registered at ClinicalTrials.gov (NCT04589884) and approved by the local ethics committee of the Faculty of Medicine of the University of Strasbourg (ID-RCB: 2020-A01896-33). A single-institution, one-arm prospective observational study was performed for this portion of the study.

### 2.2. Study Population

Adult patients undergoing a major hepatic resection (4 or more segments) through an open approach between 1 September 2020 and 30 June 2021 for malignant hepatic lesions at Nouvel Hôpital Civil (Strasbourg, France), a tertiary urban referral center, were included. Patients were eligible if the liver lesions were primary or metastatic adenocarcinoma. Patients were excluded if under 18 years of age, if the lesions were benign, if undergoing a liver biopsy or smaller resection (less than 4 segments), hepatic resection through an approach other than open laparotomy, or if the procedure was aborted prior to the experimental portion.

### 2.3. Surgical Procedure

The hepatectomy followed a standard protocol. Two experienced hepatobiliary surgeons performed all cases. In short, a laparotomy was made with a J-shaped Makuuchi incision and full exploration of the abdominal cavity was performed to look for carcinomatosis or other pathology. The liver was mobilized according to the type of hepatectomy per standard of care. The hepatic pedicle was encircled with a loop for vascular control and intermittent clamping. In healthy livers, the hepatic pedicle intermittent clamping was standardized as 20 min clamping followed by 10 min off. Whereas for cirrhotic livers, intermittent clamping consisted in 10 min of clamping followed by 10 min off [24]. Intra-operative ultrasound was performed to confirm the surgical strategy and to define the anatomical landmarks before transection. The portal vein and hepatic artery branches were ligated prior to hepatectomy. Hepatic vein ligation was not routinely performed. The transection line was scored with electrocautery once devascularization was completed. Hepatectomy was performed using the Cavitronic Ultrasonic Surgical Aspirator (CUSA, Integra Lifesciences Corporation, Plainsboro, NJ, USA). Hemostasis and biliostasis was achieved with 5-0 or 6-0 polypropylene stitches and Hem-o-lock clips. A final ultrasonography was performed to ensure hepatic inflow and outflow. External biliary drainage was left according to the type of hepatectomy and surgeon preference; drainage was not systematically used. The same standardized enhanced recovery pathway was used on all patients post-operatively [25].

### 2.4. Hyperspectral Imaging (HSI)

**Figure 1:**
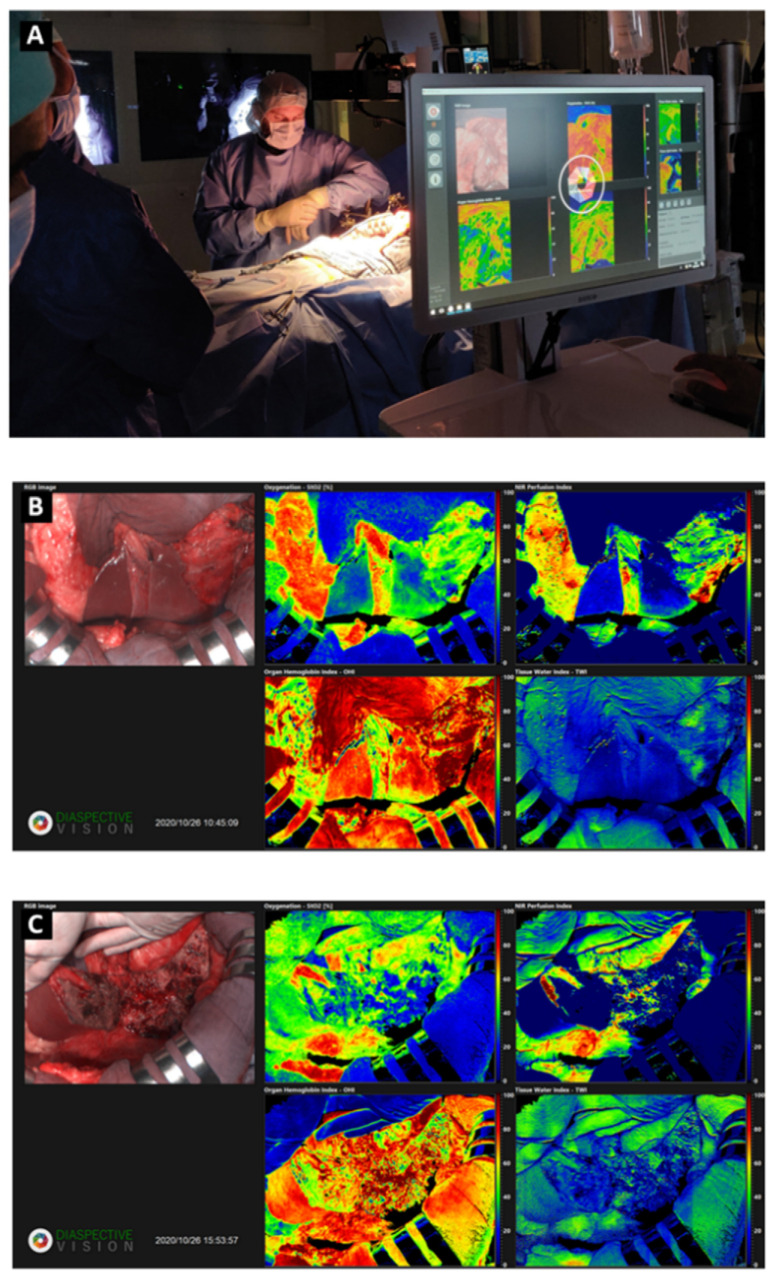
Intra-operative setting of HSI and acquired images. During the acquisition of data, all the lights of the operating room are turned off (**A**). The acquisition was performed at the beginning of the surgical procedure (**B**) and after the liver resection, at the end of surgery (**C**). The RGB (Red-Green-Blue) images and StO_2_%, NIR, OHI, and TWI indexes are reported.

The overhead light sources in the operating room were switched off during HSI acquisition. The HSI camera system (TIVITA, Diaspective Vision GmbH, Am Salzhaff, Germany) acquired hypercube (640 × 480 × 100 each) and routine RGB images for ten defined phases during the same acquisition mechanism. The HSI camera is equipped with a pushbroom imaging spectrometer with a slit-shaped aperture (motion that occurs for an HSI system to scan the field of view and acquire spectral and spatial information), an internal stepper motor controlling the slit of the spectrograph (device that breaks up a single full rotation into a number of much smaller part-rotations, mechanically connected to a diffraction grating to easily change the wavelength in the spectrograph), a high performance infrared (IR)-enhanced complementary metal oxide semiconductor (CMOS) sensor (electronic chip that converts photons to electrons for digital processing), and data processing equipment. Each hypercube was acquired in 6 s. The TIVITA hyperspectral camera was perpendicularly adjusted to a 40 cm distance from the surgical surface [26]. The system illuminates the area of interest with six halogen spotlights. The acquisition of a single hypercube was performed with a camera-specific module of the Perception Studio software (Perception Park GmbH, Graz, Austria). The spectral range of this camera is 500–995 nm. The light source per spot is a 20 W OSRAM Halospot 70 Halogen lamp allowing for intense, broadband, temperature-stable, homogeneous, and fast pulses of radiation. The calibration of the wavelength was performed during camera production. Dark current effects were corrected after the recording of the data cube by the dedicated software component. The camera collects and processes the information from the electromagnetic spectrum, measuring the reflectance spectra generated by the target of study. To convert image data from radiance to relative reflectance, a white reference object with a high diffuse reflectance is used to create a reference cube. The TIVITA^®^ camera system has preset algorithms, which can quantify the relative oxygen saturation (StO_2_%) of the superficial microcirculation at a depth up to 1 mm and the deeper layers within the near-infrared (NIR) spectrum with a penetration depth of 4–6 mm. The tissue water index (TWI) and the organ hemoglobin index (OHI) can be used to quantitatively assess and image the distribution of water and hemoglobin, respectively, in the observed region of interest (ROI) [27]. The intra-operative setting of HSI and acquired images is shown in Figure 1.

### 2.5. Data Collection

Pre-operative, intra-operative, and post-operative data were collected in a prospectively maintained electronic database. Pre-operative demographic data included gender, age, body mass index (BMI), comorbidities of dyslipidemia, diabetes mellitus (DM), hypertension, pre-existing hepatopathy, pre-operative procedures (chemoembolization, portal vein embolization, and biliary drainage), neoadjuvant chemotherapy, and American Society of Anesthesiologists classification (ASA) [28]. Laboratory values total bilirubin, aspartate transaminase (AST), alanine transaminase (ALT), gamma-glutamyl transferase (GGT), alkaline phosphatase (ALP), albumin, hemoglobin, prothrombin time (PT), and international normalized ratio (INR) were collected pre-operatively and post-operatively on post-operative days (POD) 1, POD 2, and POD 5. Intra-operative data included operative time (from skin incision to closure), intra-operative blood loss, need for transfusion, number of resected segments, clamping/ Pringle maneuver (yes/no), total duration of liver ischemia, and RGB and HSI images, with their quantitative parameters. Image acquisition was done before any liver manipulation (baseline, T0) and at the end of hepatic resection/ after specimen removal (T1). The ROIs were identified by surgeons and data scientists together and the procedure was standardized as follows: starting from the last picture (T1), the entire surface of the remaining unresected liver was considered as ROI-1; ROI-0 was exactly the same area on the liver surface at the beginning of the operation (T0). StO_2_%, NIR, TWI, and OHI values were recorded for the same ROIs at T0 and T1. Post-operative complications were recorded as major (Clavien-Dindo Class 3–5) and minor (Clavien-Dindo Class 1 and 2). Histologic data on steatosis, fibrosis, and cirrhosis from the final pathological report were collected.

### 2.6. Outcome Variables

The main outcome measure was to assess a relationship between the HSI measures, serum markers, and short-term post-operative clinical outcomes. The absolute values of StO_2_%, NIR, TWI, and OHI values were analyzed at T0 and T1. Following this, normalized values obtained via subtraction of T1 and T0 measurements (Δ) were used to correlate HSI values and surgical outcomes. Short-term outcome measures analyzed included blood loss, post-hepatectomy liver failure (PHLF), post-hepatectomy hemorrhage (PHH), bile leakage, and re-operation (unplanned return to the operating room within 60 days of the index procedure). These were defined and graded per the latest International Study Group of Liver Surgery (ISGLS) classifications [29,30,31]. The prothrombin time (PT) < 50 and serum bilirubin >50 μmol/L on POD 5 were analyzed (“50–50 criteria”) as markers of liver failure and death after major hepatectomy [32].

### 2.7. Statistical Analysis

Categorical data were expressed as frequency and percentages. Continuous variables were described as medians, with interquartile range (IQR), and compared using the Student’s t-test or the Mann–Whitney U-test, as appropriate. Normality of data distribution was assessed using histogram distribution visual inspection. Two-tailed *p*-values were considered significant when alpha was less than 0.05. Statistical analyses were performed using SPSS^®^ for Windows, v28.0 (IBM Corporation, Armonk, NY, USA).

### 2.8. Ethical Statement

Informed consent was obtained from all subjects involved prior to participation in the study. This study was part of the iEXMachyna3 project (Intraoperative EXamination Using MAChine-learning-based HYperspectral for diagNosis & Autonomous Anatomy Assessment), approved by the local ethics committee of the Faculty of Medicine of the University of Strasbourg (ID-RCB: 2020-A01896-33). The study was designed in accordance with the Declaration of Helsinki and the STrengthening the Reporting of OBservational studies in Epidemiology statement (STROBE) guidelines [33].

## 3. Results

### 3.1. Preoperative Variables

During the study period, 15 patients undergoing major hepatectomies for malignant liver tumors met inclusion criteria and were included for experimental HSI analysis. The most frequent pre-operative diagnosis was hepatocellular carcinoma (HCC, 40%), followed by colorectal cancer liver metastasis (CRLM, 33%). Five patients underwent neoadjuvant chemotherapy, and one patient had biliary drainage for pre-operative obstructive jaundice. Full demographic details and biochemical assessment are reported in Table 1.

### 3.2. Intraoperative and Postoperative Outcomes

Intra-operatively, the median operative time was 382 min. A Pringle maneuver was performed in 10 patients (67%), with a median duration of vascular intermittent clamping of 53 min. Post-operatively, eight patients total had complications (53.3%); five total were major complications. Four patients (26.9%) had liver failure, with one requiring invasive treatment (grade C PHLF). Two patients developed post-operative bile leak necessitating percutaneous drainage, one had acute renal failure requiring temporary hemodialysis, and the other had an intra-abdominal abscess that required percutaneous drainage. The overall mortality was 13.5% (*n* = 2). One died from aspiration pneumonia, septic shock and multiorgan failure (POD 21). The other from hemorrhagic shock due to hepatic artery rupture (POD 1). Full intra-operative and post-operative details are reported in Table 2.

### 3.3. Correlation between HSI and Perioperative Variables

The StO_2_% values after liver resection were significantly higher in patients who underwent pre-operative biliary drainage for jaundice (0.772 vs. 0.491, *p* = 0.033) and lower in cases with unhealthy (fatty, fibrotic, or cirrhotic) liver (0.308 vs. 0.545, *p* = 0.011). Cirrhotic liver presented higher negative ΔStO_2_% values when compared to healthy liver (−0.223 vs. 0.068, *p* = 0.05).

For the correlation between HSI and outcome variables, StO_2_% showed a significant negative correlation with ALT values on POD 5 (*r* = −0.602, *p* = 0.030), while ΔStO_2_% showed a positive correlation with ALP measured on POD 1 (*r* = 0.594, *p* = 0.032). NIR measurement at the end of operation presented higher values in unhealthy as compared to healthy liver (0.249 vs. 0.021, *p* = 0.003). This relationship was maintained when comparing fibrotic and healthy liver (0.439 vs. 0.155, *p* = 0.028). Pre-operative history of dyslipidemia was associated with lower values of final NIRS (0.072 vs 0.296, *p* = 0.029). Final NIR values showed a negative correlation with ALT values on POD 2 (*r* = −0.666, *p* = 0.013) and POD 5 (*r* = −0.696; *p* = 0.008), while intra-operative blood loss was negatively correlated with the ΔNIR (*r* = −0.629, *p* = 0.021).

TWI was the only HSI index correlated with the rate of post-operative reinterventions. The two patients who presented with biliary leakage and subsequent re-operations showed significantly lower values of final TWI (0.133 vs. 0.270, *p* = 0.038). Pre-operative chemoembolization showed significant correlation with higher final values of TWI and ΔTWI (*p* = 0.027 and *p* = 0.036, respectively). A negative correlation was found between ΔTWI and PT measured on POD 1 (*r* = −0.567, *p* = 0.043).

Lower OHI final values were significantly correlated with post-operative sepsis (0.641 vs. 0.705, *p* = 0.045), while ΔOHI was correlated with pre-operative hypertension (−0.125 vs. −0.019, *p* = 0.013) and PHLF (−0.075 vs 0.023, *p* = 0.010), as well as a negative correlation with ALT values on POD 5 (*r* = −0.813, *p* = 0.001). Full details on the relationships between HSI and peri-operative outcomes are reported in Table 3 and Table 4 and Tables S1–S4.

## 4. Discussion

The present results demonstrate the safety and feasibility of hyperspectral imaging in major hepatectomy, the ability of HSI to precisely and accurately localize ischemic damage during surgery, and the relationship of HSI measures to clinical outcomes and serum markers after this high-risk procedure. From this first human clinical application in hepatectomy, results suggest that HSI is a promising tool which could potentially help translate intra-operative advanced vision to improve short-term post-operative outcomes.

Published work has demonstrated the benefits of intra-operative evaluation of the liver parenchyma. Currently, evaluation of the remnant liver mainly relies on subjective and operator-dependent methods, naked-eye estimation, or ultrasound evaluation. This subjective evaluation becomes more challenging when underlying liver disease is present. The need for an objective, precise, and convenient analysis tool has driven researchers to find alternative solutions. ICG-fluorescence imaging is the most common objective method used to identify segmental boundaries of the liver, for more accurate anatomic resections [34,35]. Several works reported the benefit of ICG as a predictive factor of PHLF [6,36,37]. However, these studies were biased by high heterogeneity of the included population and their retrospective design. In addition, the ICG excretion rate is affected by the functional excretion ability of hepatocytes, hepatic blood flow, shunt volume, and bile flow rate, all which are unreliable in liver dysfunction [38].

HSI has great potential to overcome these limitations. Our group has previously used HSI to assess StO_2_% and intra-operatively localize preselected ROIs during esophagectomy [39], small bowel ischemia [40], and hepatectomy [18]. When adding an augmented reality function, HSI was successfully able to intra-operatively guide anatomical liver resections [18] and to discriminate between total and arterial liver ischemia [20]. The current work adds to the literature’s positive validation of prior pre-clinical studies. Promising relationships with HSI parameters and post-operative outcomes were demonstrated, as well as the relationship between prognostic laboratory values and HSI parameters.

As expected, StO_2_% had lower values in unhealthy liver tissue, as it primarily measures oxygenation close to the surface. In addition, NIR spectroscopy showed higher values in unhealthy liver tissue, especially in the presence of fibrosis. This validated the ability of HSI to measure deeper into the tissue (4–6 mm) and provided information about oxygen perfusion differences at different depths. HSI parameters of TWI and OHI were parameters significantly correlated with clinical post-operative outcomes. Specifically, the lower concentration of water inside hepatic tissue at the end of the operation was related to post-operative PHH, biliary leakage, and re-intervention. It is likely this reflects the extensive use of vascular inflow clamping and goal-directed fluid therapy with the aim to reduce intra-operative bleeding, especially in patients who underwent major hepatectomies. In support, hemoglobin concentration recognized by OHI index is affected by Pringle maneuver and a negative ΔOHI% is expected when vascular clamping is performed. Consequently, higher negative ΔOHI% reflects major reductions in hemoglobin and increased liver damage, with the resultant correlation with post-operative liver failure. Furthermore, the negative significant correlation of HSI parameters and ALT in POD 1 and POD 5 adds support to our results, as ALT is the most specific laboratory biomarker of liver injury [41].

We recognize several limitations in this study. HSI technology requires further technological advances for video-rate and time necessary for acquisition. The HSI-based enhanced reality (HYPER software) was previously developed, but not clinically validated [18]. The single center observational design, small sample size, and limited number of post-operative major complications limit the results and clinical implications. Finally, HSI acquisition is feasible only in open surgery currently. Further clinical support would allow greater development of the platform and expansion into other surgical platforms.

## 5. Conclusions

In the first clinical application of HSI for major hepatectomy, relationships between the HSI parameters and post-operative outcomes variables and laboratory marker ALT were seen. These promising but preliminary results support further clinical studies with larger samples to test the discriminative ability of the platform, independent relationships with serum markers, and clinical outcomes. Predictive models based on machine learning and larger samples could be useful to assess the real clinical applicability of HSI on liver resections. With the drive towards precision surgery, there could be great benefit from HSI guidance.

## Figures and Tables

**Table 1:** Descriptive analysis of pre-operative variables.

Variable	Total Cohort *n* = 15
Gender, male (%)	10 (67)
Age, years *	69 (49; 73)
BMI, kg/m^2^ *	28.8 (26.6; 30.1)
BMI, >30 (%)	3 (20)
ASA, ≥3 (%)	9 (60)
Dislypidemia (%)	5 (33)
Diabetes (%)	6 (40)
Hypertension (%)	5 (33)
Hepatitis (HBV or HCV) (%)	2 (13.5)
Hepatocellular carcinoma (%)	6 (40)
Colorectal metastasis (%)	5 (33)
Colangiocarcinoma (%)	2 (13.5)
Other (%)	2 (13.5)
Overall comorbidities (%)	11 (73.3)
Neoadjuvant therapy (%)	5 (33)
Pre-operative chemoembolization (%)	5 (33)
Pre-operative portal vein embolization (%)	6 (40)
Pre-operative biliary drainage (%)	1 (6.7)
Pre-operative bilirubin, µmol/L *	9.4 (5.7; 10.7)
Pre-operative serum AST, U/L *	29 (23; 63)
Pre-operative serum ALT, U/L *	46 (21; 60)
Pre-operative serum ALP, U/L *	103 (73; 174)
Pre-operative serum GGT, U/L *	86 (47; 172)
Pre-operative serum Hemoglobin, g/dL*	12.8 (11.3; 13.6)
Pre-operative serum PT, sec *	87 (75; 100)
Pre-operative serum INR *	1.1 (1; 1.21)
Pre-operative serum Albumin, g/dL *	43 (41; 46)

* Expressed as median (interquartile range). BMI body mass index, ASA American Society of Anesthesiologists, AST ASpartate transaminase, ALT ALanine Transaminase, ALP Alkaline Phosphatase, GGT Gamma-Glutamyl Transferase, PT Prothrombin Time, INR International Normalized Ratio.

**Table 2:** Descriptive analysis of intra-operative and post-operative variables.

Variable	Total Cohort *n* = 15
Number of resected hepatic segments *	5 (4; 6)
Duration of surgery, minutes *	382 (324; 452)
Vascular clamping (%)	10 (67)
Total time of vascular clamping, minutes *	53 (38; 80)
Intra-operative blood loss, mL *	490 (200; 650)
Intra-operative transfusion (%)	2 (13.5)
Post-operative complications, (Dindo–Clavien) ≥ 3 (%)	6 (40)
Post-operative 90 days mortality (%)	2 (13.5)
Liver failure (%)	4 (26.9)
Grade A (%)	1 (6.7)
Grade B (%)	2 (13.5)
Grade C (%)	1 (6.7)
PHH (%)	2 (13.5)
Biliary leakage (%)	2 (13.5)
Sepsis (%)	5 (33)
30-days re-operation (%)	2 (13.5)
POD 1 serum Bilirubin, µmol/L *	25.7 (16.4; 52.1)
POD 2 serum Bilirubin, µmol/L *	24.1 (11.1; 67.1)
POD 5 serum Bilirubin, µmol/L *	25.5 (10.4; 61.6)
POD 1 serum AST, U/L *	788 (438; 1440)
POD 2 serum AST, U/L *	608 (261; 1228)
POD 5 serum AST, U/L *	75 (36; 128)
POD 1 serum ALT, U/L*	541 (380; 1047)
POD 2 serum ALT, U/L*	531 (352; 1383)
POD 5 serum ALT, U/L *	171 (79; 323)
POD 1 serum ALP, U/L *	75 (53; 106)
POD 2 serum ALP, U/L *	87 (57; 117)
POD 5 serum ALP, U/L *	94 (62; 185)
POD 1 serum GGT, U/L *	97 (57; 126)
POD 2 serum GGT, U/L *	74 (34; 112)
POD 5 serum GGT, U/L *	75 (54; 300)
POD 1 serum PT, sec *	52 (42; 67)
POD 2 serum PT, sec *	49 (32; 64)
POD 5 serum PT, sec *	50 (35; 75)
POD5 serum Bilirubin > 50 µmol/L (%)	5 (33)
POD5 serum PT < 50 sec (%)	6 (40)
50–50 criteria (%)	2 (13.5)

* Expressed as median (interquartile range). POD post-operative day, CD Clavien–Dindo, PHH post-hepatectomy hemorrhage, AST ASpartate transaminase, ALT ALanine Transaminase, ALP Alkaline Phosphatase, GGT Gamma-Glutamyl Transferase, PT Prothrombin Time.

**Table 3:** Comparisons between final hyperspectral indexes and different peri-operative features.

			TWI Final	*p*	OHI Final	*p*	StO_2_ Final	*p*	NIR Final	*p*
Pre-operative	Chemoembolization	No	0.218 ± 0.098	0.027	0.663 ± 0.052	0.085	0.513 ± 0.154	0.930	0.276 ± 0.229	0.364
		Yes	0.307 ± 0.013		0.728 ± 0.078		0.506 ± 0.091		0.106 ± 0.095	
	Biliary drainage	No	0.244 ± 0.090	0.421	0.693 ± 0.065	0.169	0.491 ± 0.112	0.033	0.217 ± 0.213	1.000
		Yes*	0.322		0.594		0.772		0.200	
	Dislypidemia	No	0.240 ± 0.103	0.583	0.673 ± 0.062	0.350	0.528 ± 0.155	0.537	0.296 ± 0.208	0.029
		Yes	0.269 ± 0.058		0.710 ± 0.078		0.480 ± 0.078		0.072 ± 0.101	
	Hypertension	No	0.191 ± 0.074	0.060	0.677 ± 0.050	0.727	0.474 ± 0.165	0.455	0.251 ± 0.274	0.797
		Yes	0.283 ± 0.081		0.691 ± 0.078		0.531 ± 0.114		0.196 ± 0.172	
Intra-operative	Healthy liver	Yes	0.265 ± 0.076	0.814	0.657 ± 0.026	0.531	0.545 ± 0.102	0.011	0.021 ± 0.006	0.003
		No	0.248 ± 0.093		0.691 ± 0.072		0.308 ± 0.118		0.249 ± 0.204	
	Blood loss †		0.357	0.211	0.365	0.199	−0.100	0.735	−0.024	0.934
Post-operative	Liver failure	No	0.247 ± 0.074	0.768	0.681 ± 0.070	0.566	0.517 ± 0.140	0.738	0.198 ± 0.207	0.368
		Yes	0.262 ± 0.153		0.707 ± 0.067		0.487 ± 0.110		0.282 ± 0.225	
	Liver hemorrhage	No	0.270 ± 0.077	0.038	0.690 ± 0.073	0.664	0.513 ± 0.134	0.884	0.214 ± 0.203	0.923
		Yes	0.133 ± 0.061		0.666 ± 0.005		0.498 ± 0.156		0.228 ± 0.306	
	Bile leak	No	0.270 ± 0.077	0.038	0.690 ± 0.073	0.664	0.513 ± 0.134	0.884	0.214 ± 0.203	0.923
		Yes	0.133 ± 0.061		0.666 ± 0.005		0.498 ± 0.156		0.228 ± 0.306	
	Sepsis	No	0.270 ± 0.082	0.190	0.705 ± 0.071	0.045	0.516 ± 0.074	0.826	0.235 ± 0.213	0.635
		Yes	0.200 ± 0.096		0.641 ± 0.034		0.498 ± 0.240		0.168 ± 0.204	
	Re-operation	No	0.270 ± 0.077	0.038	0.690 ± 0.073	0.664	0.513 ± 0.134	0.884	0.214 ± 0.203	0.923
		Yes	0.133 ± 0.061		0.666 ± 0.005		0.498 ± 0.156		0.228 ± 0.306	
	ALP POD1 †		−0.200	0.492	0.007	0.982	0.443	0.113	0.137	0.642
	PT POD1 †		−0.150	0.609	0.253	0.382	−0.026	0.929	0.011	0.970
	ALT POD2 †		−0.325	0.279	−0.127	0.680	−0.517	0.070	−0.666	0.013
	ALT POD5 †		−0.191	0.531	−0.004	0.989	−0.602	0.030	−0.696	0.008

* only 1 case in the present population. † Spearman’s rank correlation coefficient. POD post-operative day, ALP Alkaline Phosphatase, PT Prothrombin Time, ALT ALanine Transaminase.

**Table 4:** Comparisons between differential values of hyperspectral indexes and different peri-operative features.

			ΔTWI	*p*	ΔOHI	*p*	ΔStO_2_	*p*	ΔNIR	*p*
Pre-operative	Chemoembolization	No	−0.049 ± 0.077	0.036	−0.064 ± 0.083	0.798	0.024 ± 0.174	0.545	−0.001 ± 0.241	0.435
		Yes	0.059 ± 0.083		−0.052 ± 0.082		0.078 ± 0.092		−0.088 ± 0.109	
	Biliary drainage	No	−0.016 ± 0.093	0.280	−0.060 ± 0.083	0.966	0.024 ± 0.130	0.067	−0.054 ± 0.195	0.368
		Yes *	0.093		−0.056		0.299		0.199	
	Dislypidemia	No	−0.001 ± 0.119	0.773	−0.071 ± 0.070	0.527	0.037 ± 0.168	0.820	−0.039 ± 0.250	0.943
		Yes	−0.018 ± 0.039		−0.041 ± 0.098		0.057 ± 0.119		−0.027 ± 0.099	
	Hypertension	No	−0.032 ± 0.092	0.484	−0.125 ± 0.057	0.013	0.075 ± 0.150	0.579	−0.052 ± 0.266	0.943
		Yes	0.007 ± 0.098		−0.019 ± 0.064		0.026 ± 0.150		−0.024 ± 0.165	
Intraoperative	Healthy liver	Yes	0.065 ± 0.092	0.063	−0.036 ± 0.035	0.879	0.037 ± 0.225	0.841	−0.177 ± 0.165	0.064
		No	−0.013 ± 0.078		−0.030 ± 0.091		0.022 ± 0.123		−0.032 ± 0.146	
	Blood loss †		0.222	0.467	0.507	0.077	−0.427	0.146	−0.629	0.021
Post-operative	Liver failure	No	−0.020 ± 0.077	0.263	−0.075 ± 0.077	0.010	0.069 ± 0.133	0.171	−0.004 ± 0.201	0.154
		Yes	0.063 ± 0.186		0.023 ± 0.022		−0.087 ± 0.191		−0.202 ± 0.067	
	Liver hemorrhage	No	−0.006 ± 0.098	0.861	−0.048 ± 0.072	0.090	0.029 ± 0.141	0.203	−0.038 ± 0.207	1.000
		Yes *	−0.024		−0.189		0.228		0.011	
	Bile leak	No	−0.006 ± 0.098	0.861	−0.048 ± 0.072	0.090	0.029 ± 0.141	0.203	−0.038 ± 0.207	1.000
		Yes *	−0.024		−0.189		0.228		0.011	
	Sepsis	No	−0.018 ± 0.102	0.474	−0.045 ± 0.079	0.246	0.019 ± 0.115	0.257	−0.022 ± 0.167	1.000
		Yes	0.028 ± 0.060		−0.108 ± 0.071		0.132 ± 0.231		−0.077 ± 0.329	
	Re-operation	No	−0.006 ± 0.098	0.861	−0.048 ± 0.072	0.090	0.029 ± 0.141	0.203	−0.038 ± 0.207	1.000
		Yes *	−0.024		−0.189		0.228		0.011	
	ALP POD1 †		0.146	0.635	−0.239	0.431	0.594	0.032	0.160	0.603
	PT POD1 †		−0.567	0.043	0.110	0.720	−0.234	0.442	0.267	0.378
	ALT POD2 †		0.266	0.404	0.140	0.665	0.049	0.880	−0.154	0.633
	ALT POD5 †		−0.105	−0.745	−0.813	0.001	−0.091	0.778	−0.312	0.324

* only 1 case in the present population. † Spearman’s rank correlation coefficient. POD post-operative day, ALP Alkaline Phosphatase, PT Prothrombin Time, ALT ALanine Transaminase.

## Data Availability

Data presented in this study are available in the article and Supplementary Materials.

## Supplementary Materials

The following supporting information can be downloaded at: https://www.mdpi.com/article/10.3390/cancers14225591/s1, Table S1: Comparison of final hyperspectral indexes between patients with different perioperative features; Table S2: Comparison of differential values of hyperspectral indexes between patients with different perioperative features; Table S3: Correlation between final hyperspectral indexes and perioperative continuous variables; Table S4: Correlation between differential hyperspectral indexes and perioperative continuous variables.
