# Supplementary material for: Hyperspectral Imaging in Major Hepatectomies: Preliminary Results from the Ex-Machyna Trial
Source: Cancers (Basel). 2022 Nov 14;14(22):5591. doi: 10.3390/cancers14225591 (PMC9688371; doi:10.3390/cancers14225591)
Supplement: Supplementary file 1 [file cancers-14-05591-s001.zip › cancers-1875150-supplementary.pdf]

Supplementary Materials

Table S1 Comparison of final hyperspectral indexes between patients with different perioperative features

Table S2 Comparison of differential values of hyperspectral indexes between patients with different perioperative features

Table S3 Correlation between final hyperspectral indexes and perioperative continuous variables

Table S4 Correlation between differential hyperspectral indexes and perioperative continuous variables

Table S1 Comparison of final hyperspectral indexes between patients with different perioperative features

		TWI final	*p*	OHI final	*p*	StO_2 final	*p*	NIR final	*p*
**BMI**	**< 30**	0.239 ± 0.086	.384	0.680 ± 0.070	.520	0.538 ± 0.135	.154	0.237 ± 0.209	.555
	**≥ 30**	0.291 ± 0.104		0.710 ± 0.065		0.413 ± 0.041		0.137 ± 0.206	
**Pre-existing hepatopathy**	**No**	0.235 ± 0.085	.118	0.682 ± 0.066	.614	0.508 ± 0.139	.840	0.200 ± 0.217	.440
	**Yes**	0.342 ± 0.053		0.710 ± 0.105		0.529 ± 0.096		0.308 ± 0.095	
**Neoadjuvant chemotherapy**	**No**	0.263 ± 0.089	.501	0.668 ± 0.057	.192	0.498 ± 0.157	.650	0.188 ± 0.178	.699
	**Yes**	0.227 ± 0.094		0.719 ± 0.079		0.534 ± 0.075		0.265 ± 0.262	
**Preoperative portal embolization**	**No**	0.234 ± 0.090	.459	0.673 ± 0.048	.429	0.454 ± 0.120	.057	0.233 ± 0.249	1.000
	**Yes**	0.271 ± 0.090		0.704 ± 0.090		0.587 ± 0.111		0.192 ± 0.147	
**Diabetes**	**No**	0.230 ± 0.105	.272	0.696 ± 0.080	.496	0.517 ± 0.158	.829	0.263 ± 0.218	.240
	**Yes**	0.286 ± 0.028		0.669 ± 0.041		0.500 ± 0.076		0.130 ± 0.166	
**Overall comorbidities**	**No**	0.195 ± 0.085	.147	0.679 ± 0.057	.816	0.496 ± 0.182	.796	0.311 ± 0.276	.304
	**Yes**	0.272 ± 0.084		0.689 ± 0.074		0.517 ± 0.117		0.177 ± 0.172	
**Intraoperative transfusion**	**No**	0.260 ± 0.081	.333	0.685 ± 0.072	.843	0.503 ± 0.139	.609	0.213 ± 0.203	1.000
	**Yes**	0.191 ± 0.144		0.696 ± 0.047		0.557 ± 0.071		0.229 ± 0.304	
**Overall morbidity**	**No**	0.270 ± 0.050	.495	0.693 ± 0.080	.767	0.517 ± 0.057	.880	0.224 ± 0.243	.852
	**Yes**	0.235 ± 0.110		0.681 ± 0.063		0.506 ± 0.172		0.209 ± 0.189	
**Postoperative Bilirubine > 50 µmol/L in POD5**	**No**	0.254 ± 0.075	.956	0.696 ± 0.075	.653	0.497 ± 0.126	.747	0.167 ± 0.170	.622
	**Yes**	0.257 ± 0.120		0.677 ± 0.068		0.524 ± 0.164		0.211 ± 0.197	
**Postoperative PT < 50 sec in POD5**	**No**	0.243 ± 0.083	.726	0.696 ± 0.077	.577	0.519 ± 0.087	.758	0.188 ± 0.172	.639
	**Yes**	0.264 ± 0.115		0.671 ± 0.070		0.492 ± 0.208		0.212 ± 0.196	
**50-50 criteria**	**No**	0.239 ± 0.078	.533	0.689 ± 0.074	.837	0.482 ± 0.132	.409	0.166 ± 0.170	.283
	**Yes**	0.277 ± 0.128		0.679 ± 0.079		0.558 ± 0.168		0.261 ± 0.188	
**Healthy vs steatosic**	**Healthy**	0.247 ± 0.106	.926	0.675 ± 0.056	.599	0.473 ± 0.147	.370	0.288 ± 0.242	.268
	**Steatosic**	0.252 ± 0.081		0.695 ± 0.078		0.539 ± 0.120		0.161 ± 0.169	
**Healthy vs fibrosis**	**Healthy**	0.266 ± 0.081	.212	0.696 ± 0.074	.073	0.490 ± 0.141	.277	0.155 ± 0.169	**.028**
	**Fibrosis**	0.192 ± 0.107		0.650 ± 0.013		0.587 ± 0.028		0.438 ± 0.193	
**Healthy vs cirrhosis**	**Healthy**	0.24 ± 0.0840	.135	0.679 ± 0.064	.139	0.515 ± 0.136	.712	0.203 ± 0.208	.442
	**Cirrhosis***	0.380		0.785		0.461		0.375	
**Vascular clamping**	**No**	0.290 ± 0.072	.230	0.677 ± 0.078	.717	0.566 ± 0.120	.258	0.317 ± 0.228	.177
	**Yes**	0.228 ± 0.093		0.692 ± 0.066		0.480 ± 0.133		0.159 ± 0.180	

* only 1 case in the present population

BMI Body Mass Index, POD postoperative day, AST ASpartate transaminase, ALT ALanine Transaminase, ALP Alkaline Phosphatase, GGT Gamma-Glutamyl Transferase, PT Prothrombin Time, POD postoperative day

Table S2 Comparison of differential values of hyperspectral indexes between patients with different perioperative features

		ΔTWI	*p*	ΔOHI	*p*	ΔStO_2	*p*	ΔNIR	*p*
**BMI**	**< 30**	-0.020 ± 0.081	.405	-0.063 ± 0.071	.785	0.053 ± 0.128	.727	-0.006 ± 0.212	.257
	**≥ 30**	0.033 ± 0.141		-0.048 ± 0.123		0.017 ± 0.227		-0.131 ± 0.131	
**Pre-existing hepatopathy**	**No**	-0.002 ± 0.101	.642	-0.068 ± 0.081	.377	0.064 ± 0.132	.273	-0.023 ± 0.205	.769
	**Yes**	-0.038 ± 0.043		-0.011 ± 0.070		-0.062 ± 0.226		-0.097 ± 0.215	
**Neoadjuvant chemotherapy**	**No**	0.015 ± 0.092	.271	-0.055 ± 0.078	.827	0.052 ± 0.171	.839	-0.052 ± 0.216	1.000
	**Yes**	-0.045 ± 0.093		-0.066 ± 0.091		0.034 ± 0.111		-0.007 ± 0.189	
**Preoperative portal embolization**	**No**	-0.019 ± 0.102	.657	-0.053 ± 0.093	.769	-0.015 ± 0.144	.104	-0.055 ± 0.247	.628
	**Yes**	0.005 ± 0.091		-0.067 ± 0.067		0.116 ± 0.121		-0.010 ± 0.143	
**Diabetes**	**No**	-0.016 ± 0.085	.703	-0.085 ± 0.074	.145	0.051 ± 0.184	.854	-0.064 ± 0.241	.724
	**Yes**	0.005 ± 0.116		-0.018 ± 0.077		0.035 ± 0.063		0.011 ± 0.114	
**Overall comorbidities**	**No**	-0.034 ± 0.106	.526	-0.108 ± 0.051	.152	0.037 ± 0.142	.899	-0.068 ± 0.305	1.000
	**Yes**	0.003 ± 0.092		-0.038 ± 0.082		0.048 ± 0.155		-0.020 ± 0.154	
**Intraoperative transfusion**	**No**	-0.010 ± 0.098	.791	-0.066 ± 0.079	.294	0.043 ± 0.152	.863	-0.037 ± 0.208	1.00
	**Yes***	0.017		0.024		0.071		-0.001	
**Overall morbidity**	**No**	-0.039 ± 0.050	.284	-0.038 ± 0.072	.405	0.015 ± 0.063	.503	0.049 ± 0.165	.295
	**Yes**	0.018 ± 0.117		-0.077 ± 0.086		0.0709 ± 0.19		-0.107 ± 0.208	
**Postoperative Bilirubine > 50 µmol/L in POD5**	**No**	-0.027 ± 0.078	.207	-0.060 ± 0.079	.841	0.028 ± 0.108	.549	-0.068 ± 0.188	1.000
	**Yes**	0.048 ± 0.119		-0.050 ± 0.101		0.088 ± 0.232		-0.048 ± 0.197	
**Postoperative PT < 50 sec in POD5**	**No**	-0.040 ± 0.079	.120	-0.088 ± 0.089	.216	0.074 ± 0.115	.475	-0.013 ± 0.121	.315
	**Yes**	0.058 ± 0.112		-0.022 ± 0.054		-0.002 ± 0.230		-0.161 ± 0.268	
**50-50 criteria**	**No**	-0.033 ± 0.076	.122	-0.087 ± 0.083	.139	0.048 ± 0.129	.951	-0.067 ± 0.188	.921
	**Yes**	0.073 ± 0.133		-0.003 ± 0.048		0.041 ± 0.261		-0.068 ± 0.236	
**Healthy vs steatosic**	**Healthy**	0.011 ± 0.109	.571	-0.036 ± 0.056	.438	-0.040 ± 0.133	.094	-0.100 ± 0.280	.370
	**Steatosic**	-0.020 ± 0.088		-0.074 ± 0.092		0.098 ± 0.133		0.006 ± 0.134	
**Healthy vs fibrosis**	**Healthy**	-0.001 ± 0.100	.608	-0.056 ± 0.086	.777	0.043 ± 0.158	.946	-0.072 ± 0.187	.115
	**Fibrosis**	-0.041 ± 0.047		-0.0754 ± 0.01		0.052 ± 0.064		0.170 ± 0.163	
**Healthy vs cirrhosis**	**Healthy**	-0.002 ± 0.096	.525	-0.067 ± 0.077	.214	0.067 ± 0.127	**.050**	-0.017 ± 0.197	.282
	**Cirrhosis***	-0.068		0.038		-0.223		-0.249	
**Vascular clamping**	**No**	-0.025 ± 0.079	.615	-0.042 ± 0.072	.556	0.036 ± 0.186	.871	0.079 ± 0.211	.102
	**Yes**	0.003 ± 0.106		-0.070 ± 0.087		0.050 ± 0.129		-0.106 ± 0.165	

* only 1 case in the present population

BMI Body Mass Index, POD postoperative day, AST ASpartate transaminase, ALT ALanine Transaminase, ALP Alkaline Phosphatase, GGT Gamma-Glutamyl Transferase, PT Prothrombin Time, POD postoperative day

Table S3 Correlation between final hyperspectral indexes and perioperative continuous variables

	TWI final		OHI final		StO_2 final		NIR final	
	**ρ**	***p***	**ρ**	***p***	**ρ**	***p***	**ρ**	***p***
**BMI**	0.045	0.878	-0.138	0.638	-0.085	0.773	-0.041	0.890
**Preoperative bilirubine**	-0.048	0.869	0.048	0.869	-0.009	0.976	-0.026	0.929
**Preoperative AST**	-0.106	0.719	-0.247	0.395	0.249	0.391	0.456	0.101
**Preoperative ALT**	-0.480	0.082	-0.436	0.119	0.145	0.620	0.430	0.125
**Preoperative ALP**	-0.108	0.714	-0.011	0.970	0.359	0.208	0.130	0.658
**Preoperative GGT**	-0.029	0.923	-0.288	0.318	0.372	0.190	0.513	0.061
**Preoperative Hemoglobin**	0.220	0.450	-0.321	0.263	-0.053	0.858	0.097	0.742
**Preoperative PT**	-0.147	0.616	0.368	0.196	-0.334	0.243	-0.341	0.233
**Preoperative INR**	0.088	0.760	-0.333	0.244	0.229	0.432	0.284	0.325
**Preoperative Albumin**	-0.406	0.150	-0.326	0.255	-0.151	0.607	-0.035	0.904
**Total resected segments**	-0.135	0.646	0.042	0.887	0.246	0.396	-0.014	0.962
**Total duration of vascular clamping**	-0.295	0.306	0.104	0.724	-0.228	0.434	-0.237	0.416
**Operative time**	0.322	0.262	0.366	0.199	-0.070	0.811	0.196	0.502
**AST POD1**	-0.123	0.675	-0.075	0.799	-0.299	0.299	-0.374	0.188
**AST POD2**	-0.341	0.254	-0.132	0.667	-0.325	0.279	-0.380	0.201
**AST POD5**	0.105	0.734	-0.050	0.872	0.083	0.788	0.085	0.781
**ALT POD1**	-0.156	0.594	-0.130	0.658	-0.367	0.196	-0.165	0.573
**Bilirubin POD1**	-0.055	0.852	0.231	0.427	0.108	0.714	0.002	0.994
**Bilirubin POD2**	0.140	0.648	0.124	0.687	0.003	0.993	0.058	0.851
**Bilirubin POD5**	0.121	0.693	0.135	0.660	-0.019	0.950	0.121	0.693
**ALP POD2**	-0.237	0.436	-0.099	0.747	0.350	0.241	0.171	0.577
**ALP POD5**	-0.193	0.528	-0.019	0.950	0.284	0.347	-0.113	0.713
**GGT POD1**	-0.104	0.724	-0.335	0.241	0.304	0.290	0.359	0.207
**GGT POD2**	0.033	0.915	-0.331	0.270	0.190	0.534	0.259	0.393
**GGT POD5**	-0.083	0.789	-0.138	0.654	0.242	0.426	0.110	0.720
**PT POD2**	-0.025	0.936	0.220	0.469	-0.110	0.720	-0.174	0.571
**PT POD5**	-0.039	0.905	0.326	0.301	-0.298	0.347	-0.424	0.170

BMI Body Mass Index, AST ASpartate transaminase, ALT ALanine Transaminase, ALP Alkaline Phosphatase, GGT Gamma-Glutamyl Transferase, PT Prothrombin Time, POD postoperative day

Table S4 Correlation between differential hyperspectral indexes and perioperative continuous variables

	ΔTWI		ΔOHI		ΔStO_2		ΔNIR	
	**ρ**	***p***	**ρ**	***p***	**ρ**	***p***	**ρ**	***p***
**BMI**	0.245	0.420	0.008	0.979	0.305	0.310	0.077	0.802
**Preoperative bilirubine**	0.467	0.108	0.159	0.603	0.033	0.915	-0.231	0.448
**Preoperative AST**	0.028	0.929	-0.113	0.714	-0.250	0.409	-0.030	0.922
**Preoperative ALT**	-0.069	0.823	-0.569	0.042	0.014	0.964	0.171	0.577
**Preoperative ALP**	0.313	0.297	-0.258	0.394	0.533	0.061	0.022	0.943
**Preoperative GGT**	0.280	0.354	0.022	0.943	-0.022	0.943	0.088	0.775
**Preoperative Hb**	0.187	0.541	-0.104	734	-0.132	0.668	0.044	0.887
**Preoperative PT**	-0.304	0.312	-0.008	0.978	-0.014	0.964	0.148	0.630
**Preoperative INR**	0.271	0.371	-0.162	0.597	0.089	0.771	-0.162	0.597
**Preoperative Albumine**	-0.442	0.130	-0.287	0.341	-0.180	0.557	0.135	0.659
**Total resected segments**	0.356	0.233	-0.347	0.246	0.603	0.029	-0.073	0.811
**Total duration of vascular clamping**	0.085	0.783	0.178	0.560	-0.229	0.451	-0.490	0.089
**Operative time**	0.360	0.226	0.272	0.368	-0.366	0.219	-0.616	0.025
**AST POD1**	0.445	0.128	0.363	0.223	0.016	0.957	-0.291	0.334
**AST POD2**	0.294	0.354	0.238	0.457	0.014	0.966	-0.189	0.557
**AST POD5**	0.263	0.409	0.231	0.470	-0.546	0.066	-0.249	0.436
**ALT POD1**	0.407	0.168	-0.016	0.957	0.071	0.817	-0.269	0.374
**Bilirubine POD1**	0.516	0.071	-0.148	0.629	0.500	0.082	-0.121	0.694
**Bilirubine POD2**	0.545	0.067	-0.056	0.863	0.308	0.331	-0.210	0.513
**Bilirubine POD5**	0.494	0.103	-0.007	0.983	0.249	0.436	-0.221	0.491
**ALP POD2**	0.091	0.778	-0.119	0.712	0.543	0.068	0.294	0.353
**ALP POD5**	-0.133	0.680	0.263	0.409	0.228	0.477	0.242	0.449
**GGT POD1**	0.331	0.270	-0.248	0.414	0.328	0.274	0.292	0.333
**GGT POD2**	0.396	0.203	-0.095	0.770	0.315	0.318	0.074	0.820
**GGT POD5**	-0.189	0.557	0.462	0.131	-0.210	0.513	0.028	0.931
**PT POD2**	-0.508	0.092	0.175	0.586	-0.207	0.519	0.088	0.787
**PT POD5**	-0.509	0.110	-0.091	0.790	-0.091	0.790	0.018	0.958

BMI Body Mass Index, AST ASpartate transaminase, ALT ALanine Transaminase, ALP Alkaline Phosphatase, GGT Gamma-Glutamyl Transferase, PT Prothrombin Time, POD postoperative day
